# Development of flash-flood tolerant and durable bacterial blight resistant versions of mega rice variety ‘Swarna’ through marker-assisted backcross breeding

**DOI:** 10.1038/s41598-019-49176-z

**Published:** 2019-09-05

**Authors:** Sharat Kumar Pradhan, Elssa Pandit, Swapnil Pawar, Shaikh Yasin Baksh, Arup Kumar Mukherjee, Shakti Prakash Mohanty

**Affiliations:** 1 Crop Improvement Division, ICAR-National Rice Research Institute, Cuttack, India; 2 Plant Protection Division, ICAR- National Rice Research Institute, Cuttack, India

**Keywords:** Plant sciences, Plant biotechnology

## Abstract

Bacterial blight (BB) disease and submergence due to flash flood are the two major constraints for achieving higher yield from rainfed lowland rice. Marker-assisted backcross breeding was followed to develop submergence tolerant and durable BB resistant variety in the background of popular cultivar ‘Swarna’. Four BB resistance genes *viz., Xa4*, *xa5*, *xa13*, *Xa21* and *Sub1* QTL for submergence tolerance were incorporated into the mega variety. Foreground selection for the five target genes was performed using closely linked markers and tracked in each backcross generations. Background selection in plants carrying the target genes was performed by using 100 simple sequence repeat markers. Amongst backcross derivatives, the plant carrying five target genes and maximum recurrent parent genome content was selected in each generation and hybridized with recipient parent. Eighteen BC_3_F_2_ plants were obtained by selfing the selected BC_3_F_1_ line. Amongst the pyramided lines, 3 lines were homozygous for all the target genes. Bioassay of the 18 pyramided lines containing BB resistance genes was conducted against different *Xoo* strains conferred very high levels of resistance to the predominant isolates. The pyramided lines also exhibited submergence tolerance for 14 days. The pyramided lines were similar to the recurrent parent in 14 morpho-quality traits.

## Introduction

Rice, the queen of cereals is life for millions of global population. Rice grain is a source of carbohydrate, protein, specific oils, dietary fibre, vitamins, many minerals and other disease-fighting phyto-compounds for which, also known as golden cereal. The crop is cultivated in diverse agro-ecology starting from high elevation to below sea level. Globally, rice is cultivated in 163.2 million hectares of which approximately 45% area is under rainfed ecology with low productivity due to various abiotic and biotic stresses^1^. In India, rainfed lowland rice occupies around 16 million hectares area of which 92% is located in the eastern region of the country. Rice cultivation in rainfed lowland ecosystem is associated with major biotic and abiotic stresses that reduce the productivity. Improved varieties are essentially needed that combine high grain yield with submergence tolerance along with in-built resistance to major diseases and insect pests.

Among the biotic stresses, bacterial leaf blight causes considerable yield loss in this ecology. This disease caused by *Xanthomonas oryzae* pv. *oryzae* (*Xoo*) is also a destructive disease of rice in many rice growing countries of the world. The estimated yield loss due to attack of this disease is to the tune of 20–80% depending on location, season and variety^2–7^. Globally, 42 BB resistance genes have been reported from diverse sources^8^. Most of these resistance genes are tagged by closely linked molecular markers^3,9–14^. Future demand for staple food grain requirement is increasing in India. The country needs an incremental rice production of more than 2 million tons per year to meet the projected demand of 135–140 million tons by 2030. This production increase should be obtained under constraints like less land, less water, less labor and fewer chemicals, constant battle against new emerging pathogens and pests and possible adverse effects from climate change^15^. Therefore, breeding for durable disease resistance is the most effective, economical and environment friendly way to control the bacterial blight disease.

Flash-flood causing submergence is a major abiotic problem of yield limitation in eastern India^16–18^. Under flooding stress, rice plants face several challenges for proper growth and survival. It lowers the gas diffusion rate, inhibit uptake of oxygen and restricts anaerobic metabolism^19^. Again, turbid flood water further decreases accessibility of light causing severe reduction in photosynthesis. Under prolonged submergence, rice plants face energy shortage and nutrient deficiency. The stressed plants subsequently show decaying and finally die^20^. Therefore, a complete crop loss is expected under prolonged submergence in flood affected areas due to cultivation of susceptible varieties. A major QTL *Sub1* is very useful for conferring submergence tolerance for 12–14 days in flash-flood ecology^21–23^. Gene based markers for locating this major QTL are known for marker-assisted selection. This QTL has been successfully incorporated into many popular high yielding submergence susceptible varieties through marker-assisted backcrossing (MAB)^8,17,24,25^. Swarna-Sub1, the first example of submergence-tolerant mega variety, was released for submergence-prone areas of Odisha and Uttar Pradesh states in India. Recently, CR Dhan 801 and CR Dhan 802 possessing submergence and drought tolerance in the background of Swarna variety have been released for cultivation in India.

Large area coverage and long-term cultivation of a single variety possessing single resistance gene is likely to be knocked-down by the pathogen^6,26^. Therefore, gene pyramiding and stacking of multiple alleles/QTLs through molecular breeding need to be emphasized to overcome such resistance break-down in popular varieties. Simultaneous transfer of many resistance genes into a popular variety through conventional breeding method is very difficult. However, use of tightly linked molecular markers to resistance genes is highly useful in transferring the genes into recipient parents. The durability of BB resistance breakdown is much lower in four resistance gene combination of *Xa4*, *xa5*, *xa13* and *Xa21* in a single genetic background. Accumulation of submergence tolerance QTL, *Sub1* and BB resistance genes *Xa4*, *xa5*, *xa13* and *Xa21* in the mega variety ‘Swarna’ will be highly rewarding in near future in the eastern India due to its submergence tolerance and broad-spectrum BB resistance. Closely linked DNA markers have been used for several BB resistance genes that are widely used in marker-assisted selection^4–6,27–37^. Here, we report the successful gene stacking of four BB resistance genes, *Xa4*, *xa5*, *xa13* and *Xa21* and *Sub1* for submergence tolerance through marker-assisted selection (MAS) in the mega variety ‘Swarna’.

## Results

### Foreground and background selections in backcross progenies

**Figure 1:**
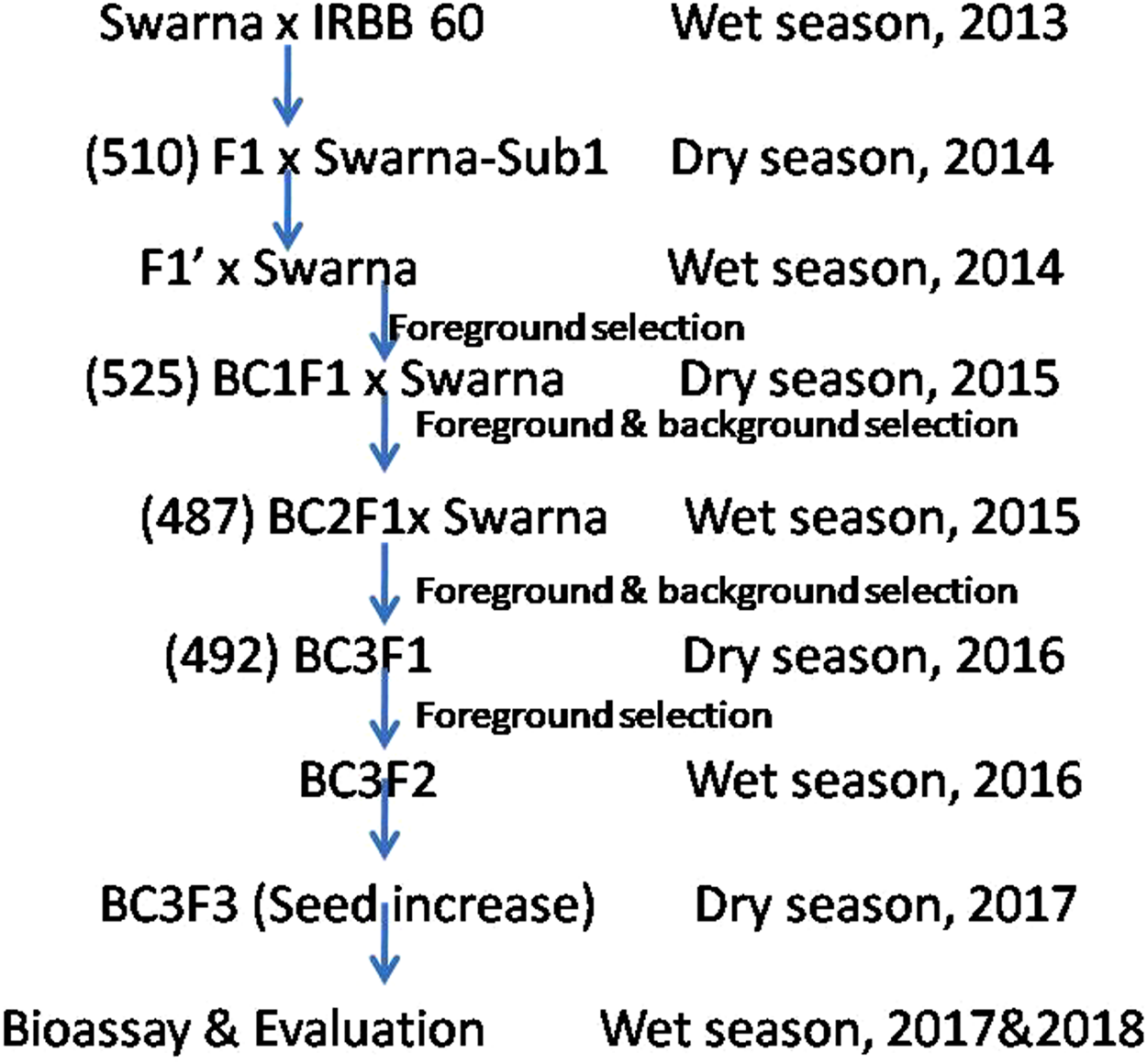
Breeding scheme used for gene stacking of *Sub1* QTL and four BB resistance genes *Xa21*, *xa13*, *xa5* and *Xa4* into variety, Swarna through marker-assisted backcrossing (Figures in parentheses indicate the number of hybrids/lines raised in that generation).

**Figure 2:**
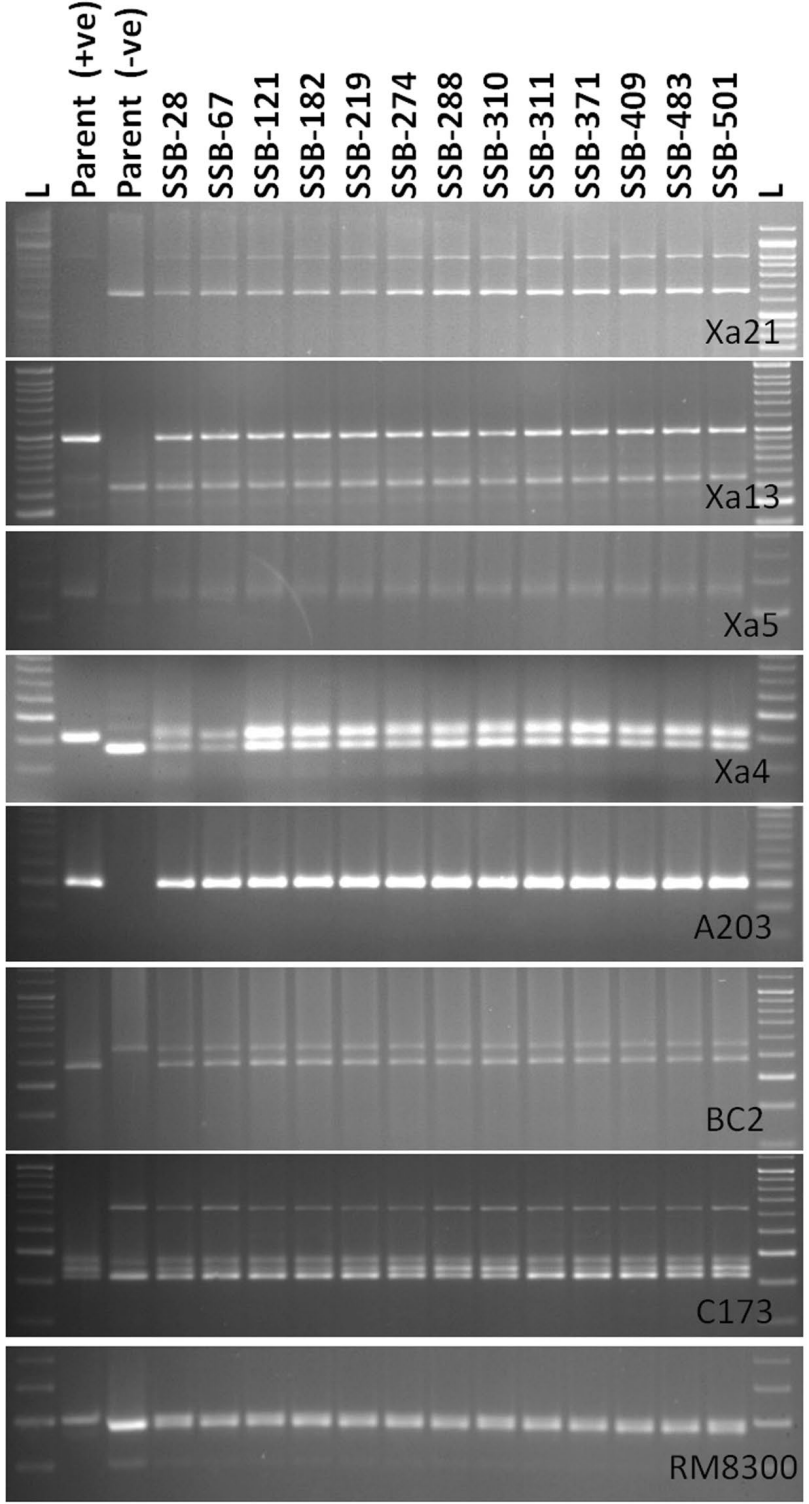
PCR amplification of markers linked to BB resistance genes *Xa21*, *xa13, xa5*, *Xa4* using primers pTA248, Xa-13 prom, RM122, MP-Nbp-131 and submergence tolerance QTL, *Sub1* using primers Sub1-A203, Sub1-BC2 Sub1-C173 and RM8300 in BC_1_F_1_ derivatives. Lanes on the top of the gel indicate the BC_1_F_1_ plants designation; L-Molecular weight marker (50 bp plus ladder).

Closely linked molecular markers for *Sub1* and BB resistance genes *Xa4*, *xa5*, *xa13* and *Xa21* were deployed for screening in each of backcross segregating generations for selecting the plants carrying five target genes (Fig. 1). True hybridity was checked in the F_1_ generation plants using the *Xa21* marker and all the plants raised from crossed seeds were found to be pure F_1_ plants. One of the pure F_1_ plants was hybridized with the third donor parent ‘Swarna-Sub1’ to combine submergence and four BB resistance genes. True multiple F_1_ plant was hybridized with the recurrent parent to produce BC_1_F_1_ seeds. The BC_1_F_1_ seeds were raised and foreground selection was performed in 525 BC_1_F_1_ plants to select plants carrying *Sub1*, *Xa4*, *xa5*, *xa13* and *Xa****2****1* genes in the lines (Fig. 2). The molecular markers used for selecting plants carrying the target genes in the derived progenies were validated first in the parental lines (Table 1). In addition, the parental polymorphism survey was performed using 1058 rice microsatellite markers covering all chromosomes, of which 100 were polymorphic that were used for background selection (Table 2).

**Table 1:** Markers used for foreground selection of *Sub1*QTL and four bacterial blight resistance genes in marker-assisted backcross breeding.

Resistance gene	Chromosome number	Marker	Primer sequences used for gene detection		Expected size (bp)	Marker type	Reference
			Forward(5′-3′)	Reverse(5′-3′)			
xa5	5	RM122	GAGTCGATGTAATGTCATCAGTGC	GAAGGAGGTATCGCTTTGTTGGAC	260 bp	SSR	^9, 11^
		xa5S (Multiplex) xa5SR/R (Multiplex)	GTCTGGAATTTGCTCGCGTTCG AGCTCGCCATTCAAGTTCTTGAG	TGGTAAAGTAGATACCTTATCAAACTGGA TGACTTGGTTCTCCAAGGCTT	160 bp	STS	^6^
xa13	8	Xa-13 prom	TCCCAGAAAGCTACTACAGC	GCAGACTCCAGTTTGACTTC	500 bp	STS	^56, 57^
Xa21	11	pTA248	AGACGCGGAAGGGTGGTTCCCGGA	AGACGCGGTAATCGAAGATGAAA	1000 bp	STS	^27^
Xa4	11	MP-Nbp-131	ATCGATCGATCTTCACGAGG	TGCTATAAAAGGCATTCGGG-	250 bp	STS	^40, 58^
Sub1	9	RM8300	GCTAGTGCAGGGTTGACACA	CTCTGGCCGTTTCATGGTAT	205 bp	SSR	^18^
		Sub1-A203	CTTCTTGCTCAACGACAACG	AGGCTCCAGATGTCCATGTC	200 bp	STS	^18^
		Sub1-BC2	AAAACAATGGTTCCATACGAGAC	GCCTATCAATGCGTGCTCTT	240 bp	STS	^18^
		Sub1-C173	AACGCCAAGACCAACTTCC	AGGAGGCTGTCCATCAGGT	170 bp	STS	^18^

**Table 2:** Microsatellite markers those are polymorphic between Swarna and IRBB60^59^.

Chromosome	No. of markers analyzed	Total No. of polymorphic markers	Name of the polymorphic markers
1	49	11	RM3148, RM7278, RM10333, RM10368, RM10505, RM3375, RM11062, RM11069, RM11229,RM11258, RM 11847
2	55	12	RM6842, RM12454, RM12601, RM13608, RM7288, RM521, RM6374, RM13249, RM13562, RM13584, RM13616, RM13702
3	44	10	RM14272, RM14603, RM4812, RM15189, RM15245, RM15379, RM15490, RM15630, RM16085, RM16238
4	46	7	RM16284, RM16396, RM16592, RM16616, RM16739, RM16903, RM17063
5	96	8	RGNMS 1946, RM18004, RM5844, RM18189, RM18384, RM18451, RM18959, RM19211
6	96	11	RM8107, RM19304, RM19623, RM19641, RM19771, RM8226/RM6836, RM527, RM20377, RM20409, RM20429, RM20773
7	96	8	RM20783, RM20847, RM336, RM21810, RM21858, RM1365, RM21976, RM22175
8	96	12	RM6369, RM152, RM22279, RM22305, RM22497, RM25, RM22521, RM22674, RM22720, RM23017, RM23377, RM23652
9	96	9	RM22431, RM219, RM23937, RM23946, RM22722, RM24616, RM24685, RM24717, RM24762
10	96	3	RM222, RM216, RM25818
11	144	5	RM26302, RM26393, RM26577, RM26824, RM26969
12	144	4	RM27552, RM27654, RM28389, RM235
Total	1058	100	

The purpose of background selection was to know the recovery of recurrent parent’s genome content in the backcross derived lines. Background selection was performed in BC_1_F_1_ to BC_3_F_2_ generations in the foreground positive plants for the five target genes. In each generation, the genotype possessing maximum genome recovery of the recurrent parent was selected for hybridization in next backcross. Background genotyping was performed using 100 SSR markers in BC_1_F_1_, BC_2_F_1_, BC_3_F_1_ and BC_3_F_2_ generations. Foreground selections in 525 BC_1_F_1_ plants showed the presence of 71 progenies carrying *Xa21* resistance gene specific bands (1000 bp), 68 plants for the presence of *xa5* resistance gene specific bands (260 bp), 84 plants with *Xa4* gene (250 bp), 58 plants carrying *xa13* (500 bp) gene while 106 plants were found to be positive for *Sub1* QTL (200 bp, 240 bp, 170 bp and 205 bp for Sub1-A203, Sub1-BC2, Sub1-C173 and RM8300). Basing on the banding pattern analysis, 46 BC_1_F_1_ plants showed the presence of *Xa21* and *xa13* resistance genes; 41 plants for the presence of *Xa21* and *xa5* resistance genes; 53 progenies showed the presence of *Xa21* and *Xa4* genes. However, we observed 15 progenies containing four target BB resistance genes *Xa21, xa13, xa5* and *Xa4*. But, out of 525 BC_1_F_1_ progenies, 13 plants were detected to be positive for all five target genes *i.e*., four target BB resistance genes and *Sub1* QTL. Background selection was performed in those 13 progenies carrying all the target genes. The recurrent genome content in those 13 lines varied from 66–81% with an average of 73.69%. The BC_1_F_1_ derived line (SSB 121) carrying highest recurrent genome content (81%) was back crossed with ‘Swarna’ to obtain BC_2_ derivatives.

**Figure 3:**
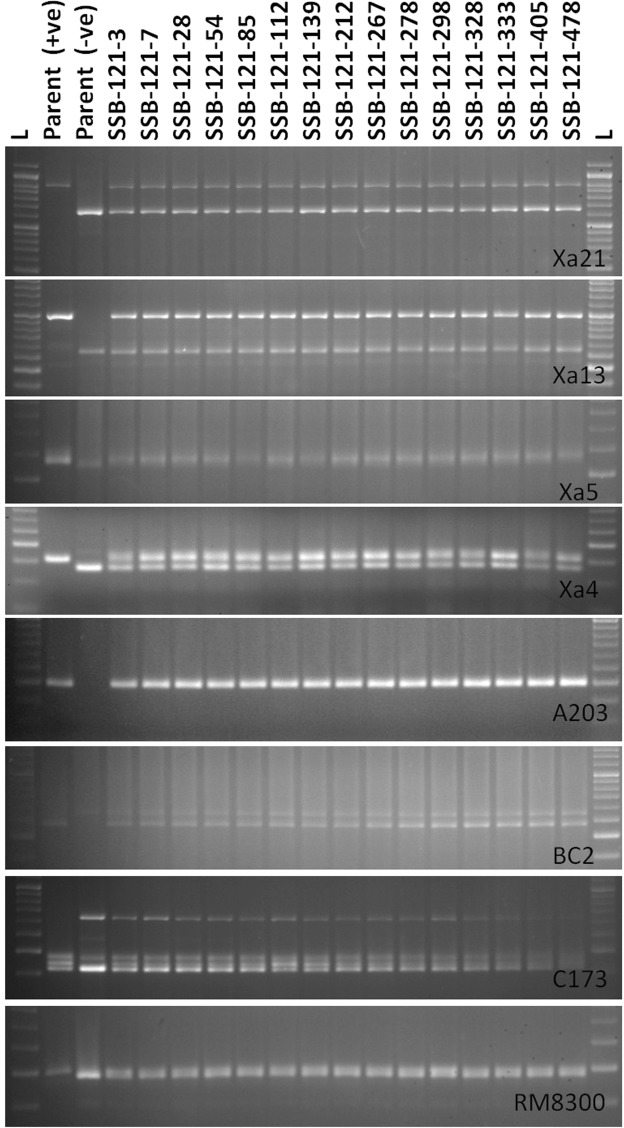
PCR amplification of markers linked to BB resistance genes *Xa21*, *xa13*, *xa5*, *Xa4* using primers pTA248, Xa-13 prom, RM122, MP-Nbp-131 and submergence tolerance QTL, *Sub1* using primers Sub1-A203, Sub1-BC2 Sub1-C173 and RM8300 in BC_2_F_1_ derivatives. Lanes on the top of the gel indicate the BC_2_F_1_ plant designation; L-Molecular weight marker (50 bp plus ladder).

Four hundred eighty seven BC_2_F_1_ plants were subjected to foreground selection by using *Sub1*, *Xa4*, *xa5*, *xa13* and *Xa21* specific markers. Fifty five, sixty one, forty eight, sixty two and eighty three progenies possessed *Xa21, xa13*, *xa5, Xa4* and *Sub1* genes, respectively. Only fifteen plants were positive for all four BB resistance genes and *Sub1* QTL (Fig. 3). Background recovery among the 15 plants with all five target genes ranged from 82–93% showing average of 88.4%. The plant (SSB 121–28) containing 93% of Swarna genome was considered for next back crossing.

**Figure 4:**
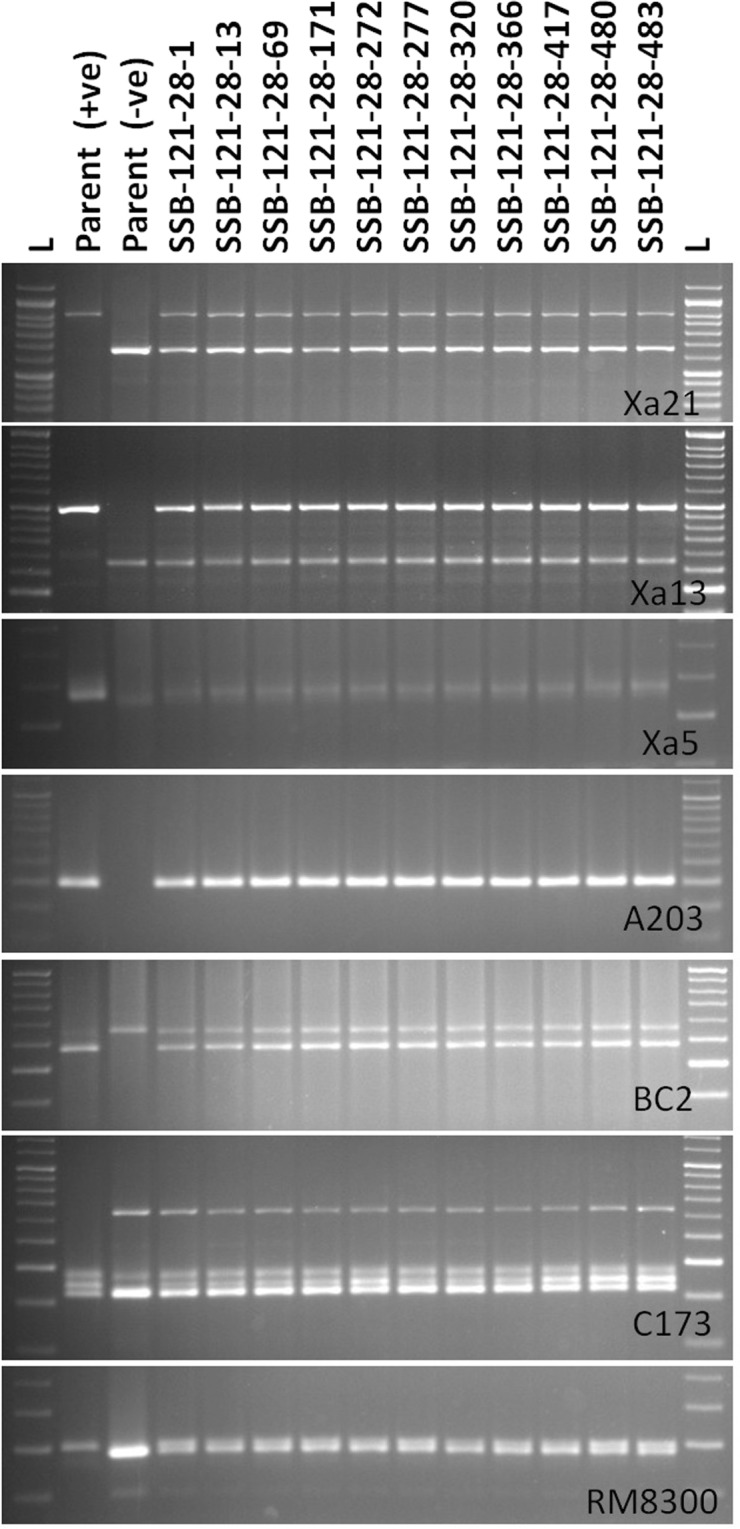
PCR amplification of markers linked to BB resistance genes *Xa21*, *xa13*, *xa5*, *Xa4* using primers pTA248, Xa-13 prom, RM122, MP-Nbp-131 and submergence tolerance QTL, *Sub1* using primers Sub1-A203, Sub1-BC2 Sub1-C173 and RM8300 in BC_3_F_1_ derivatives. Lanes on the top of the gel indicate the BC_3_F_1_ plant designation; L-Molecular weight marker (50 bp plus ladder).

**Figure 5:**
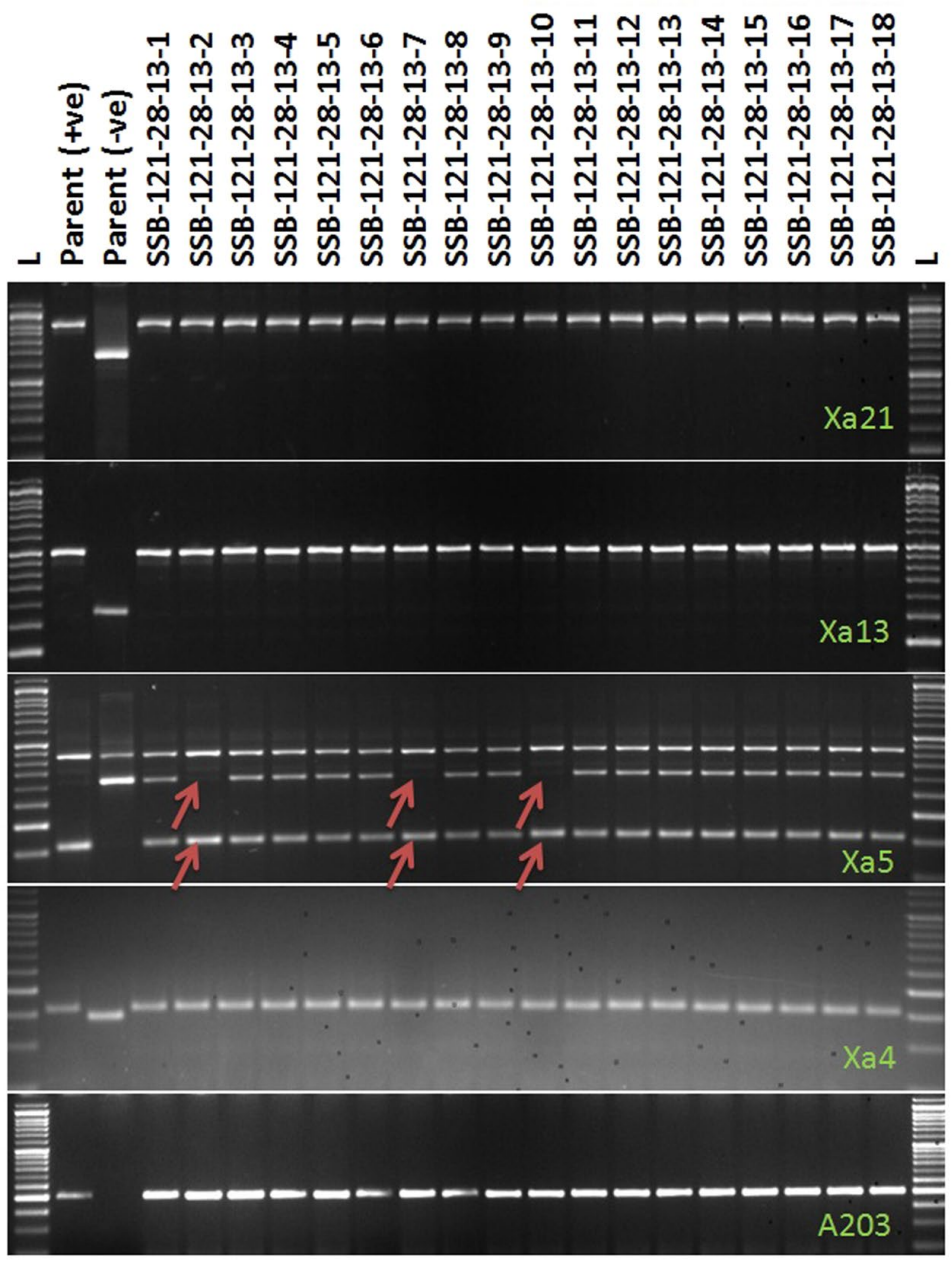
PCR amplification of markers linked to BB resistance genes *Xa21*, *xa13*, *xa5*, *Xa4* using primers pTA248, Xa-13 prom, xa5S & xa5SR/R (multiplex), MP-Nbp-131 and submergence tolerance QTL, *Sub1* using primers Sub1-A203, Sub1-BC2 Sub1-C173 and RM8300 in BC_3_F_2_ derivatives. Lanes on the top of the gel indicate the BC_3_F_2_ plant designation; L-Molecular weight marker (50 bp plus ladder).

**Figure 6:**
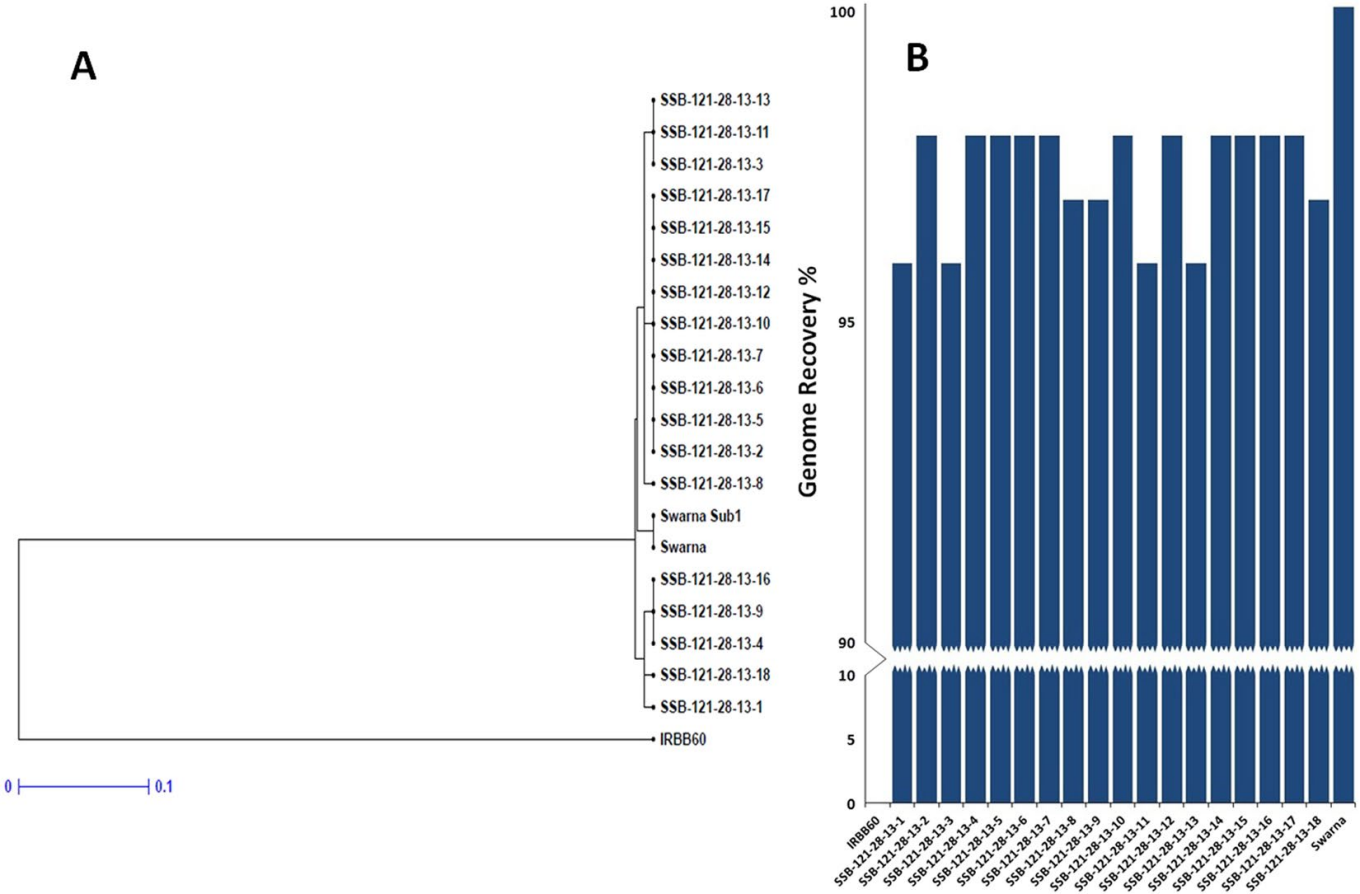
Parents and 18 pyramided lines (**A**) Dendrogram showing the genetic relationship between lines based on 100 microsatellite markers (**B**) % contribution of recurrent genome in the pyramided lines.

In BC_3_F_1_ generation, a total of 492 progenies were generated from hybridization between the BC_2_F_1_ plant (SSB 121–28) and recurrent parent ‘Swarna’. Foreground screening for five target genes was performed using the linked markers in BC_3_F_1_ progenies. A total of 63 plants positive for *Xa21*, 59 for *xa5*, 54 for *xa13* and 71 for *Xa4* were detected. When counted for BB resistance gene combinations in these progenies, 43 plants showed presence of *Xa21* and *xa13*; 38 plants with *Xa21* and *xa5* and 45 plants with *Xa21* and *Xa4*. However, 11 plants carried the target genes, *Sub1*, *Xa21*, *xa13*, *xa5* and *Xa4* (Fig. 4). The background analysis in these plants using 100 polymorphic SSR markers detected 93 to 97% recurrent genome recovery showing an average recovery of 94.27% (Table 3). During BC_3_F_2_ foreground analysis, eighteen pyramided lines were found to carry homozygous alleles of 4 target genes *viz*., *Sub1*, *Xa21*, *xa13* and *Xa4* based on gene specific markers. However, 3 plants were detected to carry all the target genes in homozygous condition by genotyping 620 BC_3_F_2_ progenies from a total of 1924 by deploying the gene specific markers (Fig. 5). Eighteen pyramided lines were evaluated for yield and other agro-morphologic traits in the BC_3_F_4_ generation. The dendrogram obtained by using the SSR data could classify the pyramided and parental lines into two major clusters (Fig. 6A). Cluster I accommodated 20 genotypes including the recipient parent ‘Swarna’, submergence tolerance donor parent ‘Swarna-Sub1’ and 18 pyramided lines while the donor parent for BB was located in cluster II. Almost all the pyramided lines in cluster I were similar in Jaccard’s coefficient value as that of the recurrent parent ‘Swarna’. Amongst the 18 BC_3_F_2_ pyramided lines, 100% similarity was noticed in SSB-121-28-13-2, SSB-121-28-13-5, SSB-121-28-13-6, SSB-121-28-13-7, SSB-121-28-13-10, SSB-121-28-13-12, SSB-121-28-13-14, SSB-121-28-13-15 and SSB-121-28-13-17 based on 100 background primers used (Fig. 6B).

**Table 3:** Number of heterozygotes identified for five target genes and estimation of recurrent parent genome contribution.

Generation	# of plants scored	# of plants that are heterozygotes for five target genes	Estimated maximum % contribution of recurrent parent genome to selected backcross plant	Average recipient parent genome content of the plants having all five gene combination	Expected % contribution of recurrent parent genome to selected backcross plants^a^
BC_1_F_1_	525	13	81	73.69	75.0
BC_2_F_1_	487	15	93	88.4	87.5
BC_3_F_1_	492	11	97	94.27	93.25

^a^As per Mendelian ratios for independent gene action.

### Screening of the pyramided lines for submergence tolerance under control condition

**Figure 7:**
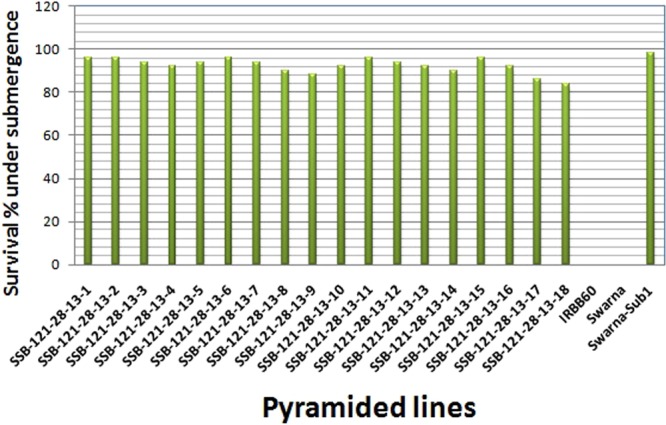
Percent plant regenerated under control screening facility using *Sub1* QTL carrying lines and its parents after one week of 14 days of submergence stress.

A total of 21 genotypes comprising 18 BC_3_F_4_ plants including 3 homozygous pyramided lines carrying *Sub1*, *Xa4*, *xa5*, *xa13* and *xa2*1 genes along with the parents were evaluated in submergence screening tank under controlled condition. A complete submergence stress of 14 days was applied to the pyramided and parental lines. All the 18 *Sub1* carrying plants exhibited regeneration ability of 84 to 96% while Swarna-Sub1 plants showed regeneration ability of 98% hills after a week of de-submergence (Fig. 7). However, no regeneration was detected in the sensitive parent IRBB60 and recipient parent Swarna. The *Sub1* pyramided lines showed almost similar regeneration ability with Swarna-Sub1 donor parent while susceptible variety IRBB60 and Swarna could not revive and thus perished completely under the submergence stress.

### Bioassays against BB disease pathogens

Eighteen pyramided lines along with 3 homozygous plants carrying 4 BB resistance genes, donor parents (IRBB60 and Swarna-Sub1) and the susceptible recurrent cultivar (Swarna) were evaluated during wet season, 2017 and 2018 after two weeks of artificial inoculation using eight *Xoo* strains for checking the BB resistance and susceptible reaction among the entries. The donor parent IRBB60 could resist the BB pathogen attack showing an average lesion length of 2.56 cm (1.8–3.1 cm) while the recurrent parent Swarna had longer average lesion length of 12.23 cm (9.8–14.0 cm) (Table 4; Supplementary Table S3). The mean lesion lengths observed in the derived lines containing *Xa21* + *xa13* + *xa5* + *Xa4* genes varied from 2.22 to 3.65 cm showing highest resistance score in SSB-121-28-13-2 (Table 4).

**Table 4:** Bacterial blight disease score and reaction of pyramided and parental lines against different *Xoo* inoculated strains pooled over wet season 2017 and 2018.

Sl. No.	Pyramided lines	Gene combination	Mean lesion length (MLL) in cm (Mean ± standard error)									
			*Xoo* strains inoculated									Disease reaction
			Xa-17	Xa-7	xa-2	xb-0.747	xc-4	xd-1	xa-1	xa-5	MLL	
1	SSB-121-28-13-1	Xa21 + xa13 + Xa4	2.5 ± 0.33	2.5 ± 0.36	3.0 ± 0.57	2.65 ± 0.7	2.9 ± 0.71	3.05 ± 0.72	2.4 ± 0.77	2.45 ± 0.81	2.68 ± 0.82	R
2	SSB-121-28-13-2	Xa21 + xa13 + xa5 + Xa4	2.95 ± 0.75	2.8 ± 0.81	2.5 ± 0.99	2.75 ± 0.86	2.75 ± 0.74	2.85 ± 0.70	2.55 ± 0.77	2.6 ± 0.90	2.72 ± 0.81	R
3	SSB-121-28-13-3	Xa21 + xa13 + Xa4	2.9 ± 0.94	2.55 ± 0.56	2.85 ± 0.94	3.05 ± 0.62	2.85 ± 0.74	2.7 ± 0.84	2.25 ± 1.08	2.25 ± 0.91	2.67 ± 0.82	R
4	SSB-121-28-13-4	Xa21 + xa13 + Xa4	2.75 ± 0.77	2.75 ± 0.89	2.9 ± 0.74	3.15 ± 0.64	3.25 ± 0.47	2.9 ± 0.79	2.85 ± 0.74	2.5 ± 0.69	2.88 ± 0.71	R
5	SSB-121-28-13-5	Xa21 + xa13 + Xa4	2.65 ± 0.69	3.15 ± 0.61	2.75 ± 0.75	2.35 ± 0.60	3.05 ± 0.59	2.5 ± 0.84	2.25 ± 1.08	2.8 ± 0.78	2.67 ± 0.74	R
6	SSB-121-28-13-6	Xa21 + xa13 + Xa4	2.15 ± 0.67	2.4 ± 0.53	2.75 ± 0.83	2.5 ± 0.63	2.6 ± 0.88	2.95 ± 0.80	3.0 ± 0.50	2.55 ± 0.83	2.61 ± 0.71	R
7	SSB-121-28-13-7	Xa21 + xa13 + xa5 + Xa4	3.05 ± 0.62	2.75 ± 0.65	2.9 ± 0.66	2.6 ± 0.82	2.65 ± 0.79	2.2 ± 0.74	3.65 ± 1.09	2.45 ± 0.5	2.78 ± 0.73	R
8	SSB-121-28-13-8	Xa21 + xa13 + Xa4	3.4 ± 0.93	3.25 ± 0.94	2.3 ± 0.89	2.5 ± 0.79	2.75 ± 0.68	2.8 ± 0.79	2.9 ± 0.76	2.8 ± 0.78	2.84 ± 0.82	R
9	SSB-121-28-13-9	Xa21 + xa13 + Xa4	3.05 ± 0.79	2.55 ± 0.64	2.7 ± 1.04	2.65 ± 0.73	3.15 ± 1.2	2.35 ± 0.85	2.75 ± 0.83	2.95 ± 0.79	2.77 ± 0.86	R
10	SSB-121-28-13-10	Xa21 + xa13 + xa5 + Xa4	3.0 ± 0.75	2.55 ± 0.67	3.15 ± 0.78	3.25 ± 0.65	2.7 ± 0.49	2.95 ± 0.79	2.8 ± 0.60	3.25 ± 0.75	2.95 ± 0.68	R
11	SSB-121-28-13-11	Xa21 + xa13 + Xa4	3.4 ± 0.99	2.55 ± 0.70	3.4 ± 0.94	3.05 ± 0.63	2.85 ± 0.64	3.0 ± 0.81	3.05 ± 0.66	3.25 ± 0.72	3.07 ± 0.76	MR
12	SSB-121-28-13-12	Xa21 + xa13 + Xa4	3.1 ± 0.67	2.9 ± 0.56	3.55 ± 0.89	3.3 ± 0.68	3.05 ± 0.60	3.1 ± 0.83	2.8 ± 0.89	2.95 ± 0.87	3.09 ± 0.75	MR
13	SSB-121-28-13-13	Xa21 + xa13 + Xa4	3.3 ± 0.87	3.3 ± 1.1	2.65 ± 0.55	2.55 ± 0.69	2.7 ± 0.68	2.95 ± 0.72	3.0 ± 0.71	2.65 ± 0.81	2.89 ± 0.77	R
14	SSB-121-28-13-14	Xa21 + xa13 + Xa4	2.5 ± 0.32	2.5 ± 0.51	2.45 ± 0.54	3.3 ± 0.72	2.35 ± 0.55	2.15 ± 0.37	3.4 ± 1.13	2.25 ± 0.54	2.63 ± 0.58	R
15	SSB-121-28-13-15	Xa21 + xa13 + Xa4	2.7 ± 0.64	2.75 ± 1.07	2.3 ± 0.73	2.65 ± 0.67	2.8 ± 0.67	3.2 ± 1.26	2.4 ± 0.53	3.85 ± 1.19	2.83 ± 0.84	R
16	SSB-121-28-13-16	Xa21 + xa13 + Xa4	3.05 ± 0.86	2.75 ± 0.72	2.75 ± 0.62	3.05 ± 0.83	3.25 ± 1.05	3.4 ± 0.81	2.5 ± 0.89	2.8 ± 0.80	2.94 ± 0.82	R
17	SSB-121-28-13-17	Xa21 + xa13 + Xa4	4.2 ± 0.69	4.25 ± 0.70	3.4 ± 0.62	3.6 ± 0.74	4.95 ± 0.85	4.6 ± 0.76	5.15 ± 0.75	4.5 ± 0.85	4.33 ± 0.74	MR
18	SSB-121-28-13-18	Xa21 + xa13 + Xa4	5.3 ± 0.81	3.6 ± 0.80	4.45 ± 0.89	4.0 ± 0.94	4.0 ± 0.75	4.45 ± 0.72	4.6 ± 0.61	4.95 ± 0.81	4.42 ± 0.79	MR
19	IRBB60 (donor)	Xa21 + xa13 + Xa4	2.05 ± 0.49	2.55 ± 0.49	2.4 ± 0.60	2.55 ± 0.47	3.0 ± 0.80	2.65 ± 0.52	2.85 ± 0.82	2.45 ± 0.55	2.56 ± 0.59	R
20	Swarna-Sub1	—	12.05 ± 1.3	12.75 ± 1.3	12.3 ± 1.25	10.9 ± 1.02	10.5 ± 1.2	10.05 ± 0.66	10.5 ± 0.88	11.5 ± 0.87	11.32 ± 1.05	S
21	Swarna(recipient)	—	13 ± 1.41	12.75 ± 1.4	12.45 ± 1.4	10.95 ± 1.2	10.05 ± 1.3	11.2 ± 1.59	13.7 ± 1.7	13.75 ± 1.6	12.23 ± 1.46	S

### Grain yield and morpo-quality traits of the pyramided lines carrying *Sub1* and BB resistance genes

**Figure 8:**
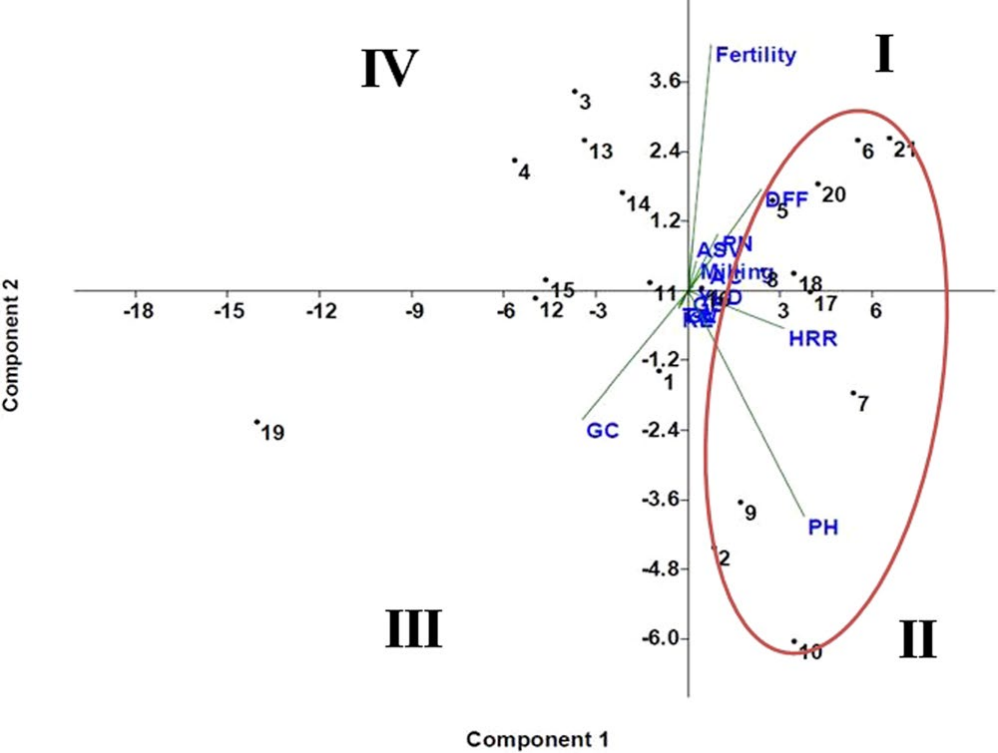
Biplot diagram of 18 pyramided lines carrying five genes (*Sub1*, *Xa21, xa13, xa5* and *Xa4*) along with parents for first two principal components. The numbers in the figure indicate the serial number of the genotypes enlisted in Table 4. PH-Plant height; DFF-Days to 50% flowering; PN- Panicles/plant; Fertility- Number of filled grains/panicle; TW-1000-grain weight; GL- Grain length; GB-Grain breadth, Milling (%); HRR- Head rice recovery (%); KE-Kernel length after cooking (mm); ASV-Alkali spreading value; GC-Gel consistency; AC- Amylose content (%) and YLD- plot yield.

Eighteen pyramided lines including 3 homozygous carrying five target genes in the background of Swarna in BC_3_F_4_ generation along with the donor and recipient parents were evaluated during wet season, 2017 at NRRI, Cuttack. The recurrent parent, Swarna produced mean grain yield of 5.35 t/ha. Many test entries with five target genes showed grain yield higher than the recurrent parent, Swarna (Table 5). Most of the pyramided lines were similar in various morpho-quality traits like the recipient parent, Swarna (Table 5). The genotype-by-trait biplot diagram generated using 14 morphologic and quality traits of the 18 pyramided lines along with parents also indicated clearly the morphologic and quality traits similarity among pyramided lines from the placement pattern in the quadrants (Fig. 8). All the pyramided lines present in the first quadrant were superior in grain quality, grain yield and other parameters similar to the popular variety, Swarna. These lines in 1^st^ quadrant and the lines present in the 2^nd^ quadrant may be considered for further evaluation and release as cultivars in the country. The first principal component explained 60.2% of variation while second component showed 15.6% of the total variation. Amongst the 14 studied morpho-quality traits, spikelet fertility and plant height contributed maximum towards diversity (Fig. 8).

**Table 5:** Agro-morphologic and grain quality parameters of BC_3_F_4_ pyramided lines along with parents under field evaluation.

Serial Number	Pyramided lines	Plan height (cm)	Days to 50% flowering	panicles/plant	Spikelet Fertility %	1000- seed weight (g)	Grain length (mm)	Grain breadth (mm)	Milling (%)	Head rice recovery (%)	Kernel elongation after cooking	Alkali spreading value	Gel consistency	Amylose content (%)	Plot yield (t/ha)
1	SSB-121-28-13-1	101	108	14.9	86.4	20.12	5.65	2.21	68.5	63.1	8.4	6.0	63	24.75	6.445
2	SSB-121-28-13-2	103	110	13.6	83.5	20.40	5.75	2.32	68.4	64.2	8.5	4.5	63	24.65	6.385
3	SSB-121-28-13-3	96	107	14.8	88.6	20.60	5.48	2.15	67.9	61.5	8.3	6.0	61	24.58	6.365
4	SSB-121-28-13-4	95	108	14.2	86.7	20.75	5.75	2.25	67.9	60.7	8.6	5.0	63	24.94	6.250
5	SSB-121-28-13-5	102	110	14.5	89.2	20.65	5.70	2.23	69.4	63.2	8.4	5.0	59	25.16	6.180
6	SSB-121-28-13-6	102	110	14.7	90.1	20.10	5.40	2.10	69.1	66.5	8.3	5.5	57	25.27	6.120
7	SSB-121-28-13-7	104	110	13.8	85.3	20.90	5.80	2.35	69.3	65.4	9.1	5.5	57	25.15	5.925
8	SSB-121-28-13-8	101	109	14.1	87.3	19.75	5.45	2.05	66.5	65.2	8.3	6.0	59	25.05	5.905
9	SSB-121-28-13-9	103	109	14.3	84.2	21.90	5.52	2.10	66.3	64.5	8.4	5.0	61	24.65	5.895
10	SSB-121-28-13-10	106	108	14.5	83.7	20.25	5.72	2.42	66.7	65.3	8.5	4.5	61	24.35	5.850
11	SSB-121-28-13-11	100	108	15.2	88.4	21.60	5.32	2.05	66.8	63.4	8.3	4.5	63	25.12	5.815
12	SSB-121-28-13-12	96	109	13.2	85.4	20.25	5.65	2.18	65.8	63.2	8.4	5.0	65	24.55	5.785
13	SSB-121-28-13-13	97	110	14.3	88.5	21.65	5.56	2.25	67.3	61.5	8.5	5.5	63	23.55	5.760
14	SSB-121-28-13-14	98	110	14.7	86.5	20.55	5.85	2.45	67.8	60.8	8.8	6.0	61	24.21	5.735
15	SSB-121-28-13-15	96	108	13.6	83.5	21.75	6.15	2.45	66.7	60.7	8.9	6.5	61	24.35	5.710
16	SSB-121-28-13-16	98	108	13.4	84.5	20.25	5.65	2.12	69.2	66.1	8.4	6.0	59	25.15	5.650
17	SSB-121-28-13-17	103	109	13.2	87.6	21.45	5.56	2.18	69.3	64.3	8.5	5.5	57	23.75	5.620
18	SSB-121-28-13-18	101	109	14.6	86.5	21.65	6.05	2.05	67.3	65.6	8.7	5.0	57	24.15	5.605
19	IRBB60 (donor)	95	101	9.8	85.2	21.15	6.35	2.08	69.1	55.1	9.9	4.5	67	22.55	4.525
20	Swarna-Sub1 (donor)	102	110	14.6	88.6	20.32	5.32	2.25	68.2	64.2	8.2	6.0	57	25.32	5.850
21	Swarna (recipient)	103	114	15.2	88.9	19.2	5.30	2.23	68.4	64.5	8.1	6.0	57	25.42	5.355
LSD_5%_		10.46	10.91	1.58	8.72	1.82	0.72	0.164	7.242	7.626	0.754	—	—	2.684	0.582
CV%		3.86	0.95	10.42	4.56	4.62	6.35	7.286	6.685	9.328	7.36	—	—	6.742	11.659

### Analysis of genome introgression on the carrier chromosomes

**Figure 9:**
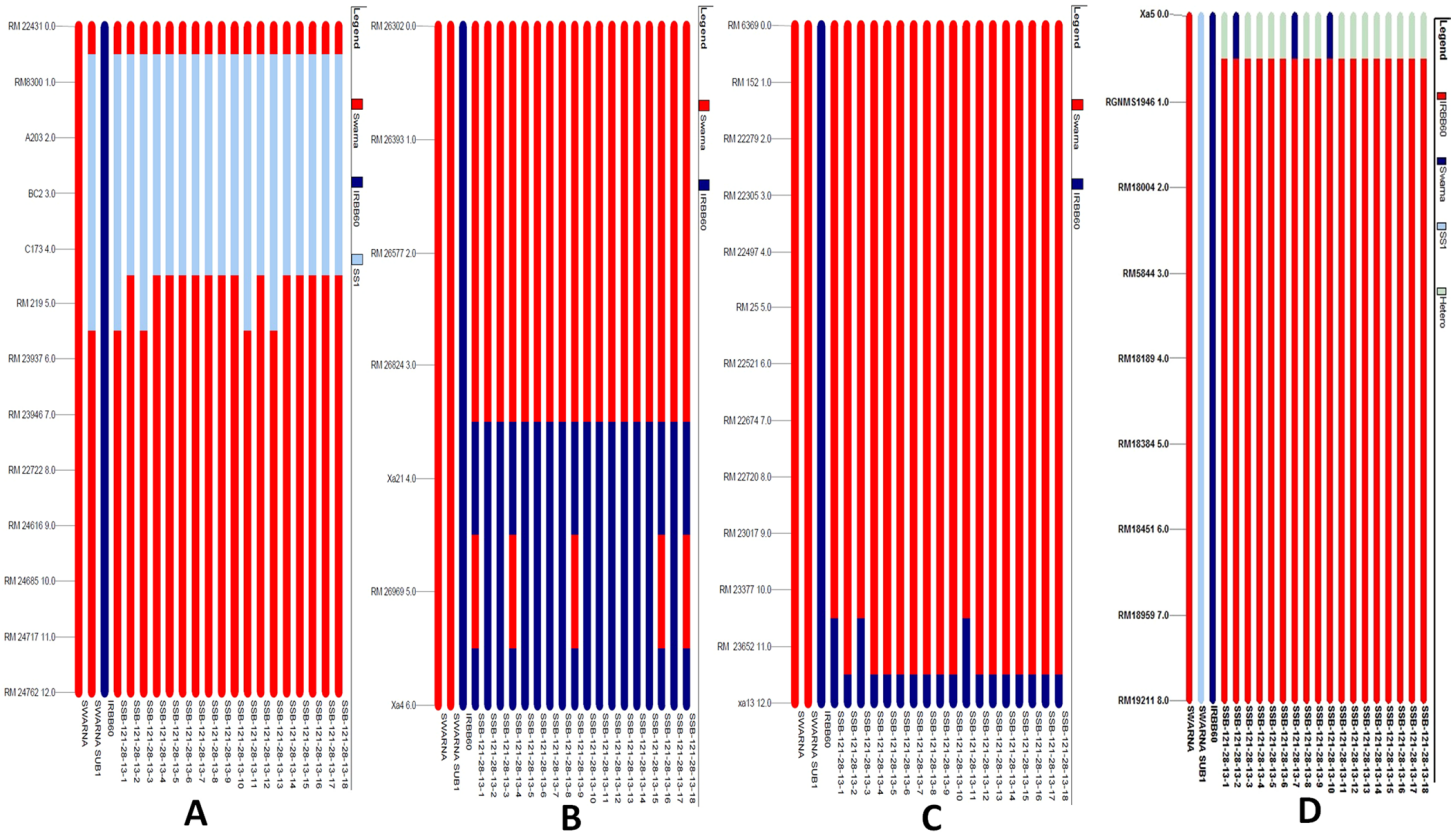
Analysis of genome recovery of 18 pyramided lines associated with submergence tolerance QTL and bacterial blight resistance genes (**A**) Sub1 QTL on chromosome 9 (**B**) *Xa21* and Xa4 on chromosome 11 (**C**) *xa13* on chromosome 8 and (**D**) *xa5* on chromosome 5 in Swarna, IRBB60 and Swarna-Sub1 BC_3_F_3_ derivatives.SS-Submergence tolerance donor parent Swarna-Sub1.

Hundred polymorphic SSR markers showing good coverage of individual chromosomes were used for analysis of recurrent genome recovery as well as presence of linkage drag, if any, in each generation. Particularly, the markers were selected carefully in the carrier chromosomes to track the linkage drag (Supplementary Table 1). The genotyping results of 18 BC_3_F_2_ derived lines showed the presence of homozygous target genes (Fig. 9). The detailed variations observed with the 100 polymorphic markers among these lines are presented in Supplementary Table 1. A linkage drag segment from donor parent was observed in *Sub1* carrier chromosome 9 in between primer C173 and RM219 in the pyramided lines SSB-121-28-13-1, SSB-121-28-13-3, SSB-121-28-13-11 and SSB-121-28-13-13 while no linkage drag was observed in rest 14 pyramided lines. The carrier chromosome 11 for *Xa21* and *Xa4*, showed no donor segment in the pyramided lines SSB-121-28-13-1, SSB-121-28-13-4, SSB-121-28-13-9, SSB-121-28-13-16 and 18 while other 14 lines were with a donor linkage drag (Fig. 9). No linkage drag segment was observed on *xa5* carrier chromosome 5 but a donor segment was observed on *xa13* carrier chromosome 8 in SSB-121-28-13-1, SSB-121-28-13-3 and SSB-121-28-13-11 pyramided lines (Fig. 9).

## Discussion

Deployment of molecular markers for selection of target gene carrying segregating genotypes in breeding programs enhances the precision of target gene transfer to the recipient variety, as well as reduces the duration of selection cycle compared to conventional breeding program. In this breeding program, we could achieve transfer of four BB resistance genes and *Sub1* QTL into mega variety, ‘Swarna’ by integrating molecular markers with the phenotypic selections. The pyramided lines were developed in three backcrosses followed by two selfing generations to achieve the desired product. Control of bacterial blight disease using resistance genes is economical and environment friendly. Again, *Sub1* QTL was introduced into the background so as to confer tolerance to submergence during flash flood stress. Therefore, it is a significant achievement in lowland rice crop improvement program by gene stacking of two important traits through molecular breeding. Development of varieties through marker-assisted selection has been reported earlier with less duration, more precision and more environment friendly approaches^4–7,27,28,34–38^. However, by adopting this precision breeding, *Sub1* QTL along with 4 BB resistance genes were stacked and few Swarna type pyramided lines suitable for the flash flood and BB endemic areas of the country could be developed.

Many successful examples of research results on gene pyramiding have been reported^4–7,27,28,34–38^. However, development of cultivar with gene stacked for broad spectrum resistance against BB pathogen and submergence tolerance in the mega variety, Swarna is clearly different from other’s results. Previous research publications on gene stacking of submergence tolerance and BB resistance in rice varieties such as improved Tapaswini and improved Lalat have been reported^39,40^. These two varieties are early to mid early type and are not suitable for lowland ecology. But, our gene stacking results are on a popular variety that is a late maturing type and hence suitable for lowland ecology. Other publications using MAS breeding are mostly pyramiding of genes for development of cultivars to insects and diseases in rice^4–7,27,28,34–40^.

The recovery of higher percentage of recipient parent genome with less drag from donor parents IRBB60 and Swarna-Sub1 using 100 polymorphic primers is also an important achievement. Similar rice breeding work for transfer of various traits have been reported earlier^5–7,14,34–37,41^. Using this molecular breeding approach, it was possible to develop plants carrying five target genes with recurrent parent genome of more than 95% in the pyramided lines. The results of this breeding program resulted in development of 18 pyramided high yielding lines homozygous for *Sub1*, *Xa21*, *xa13* and *Xa4* exhibiting maximum recurrent parent genome recovery. However, 3 pyramided lines were found to carry all five target genes. There may be a “pull” for introgression of the *Xa21*, *xa13* and *xa5* genes during selection that helps in dragging of additional unlinked loci from the donor genome in backcross generations^35^. However, we could detect such pull effects only in few pyramided lines during the transfer of *Sub1*, *Xa21*, *xa13*, *xa5* and *Xa4* genes into Swarna background in different backcross generations. The graphical genotyping data of the pyramided lines also indicated the linkage drag in the carrier chromosomes carrying the five target genes (Fig. 9). No donor segment linkage drag was seen in *xa5* carrier chromosome 5. Similar types of results were observed in earlier publications with foreground and background selections with less linkage drag by involving more numbers of background markers^5,6,35,36,42^. In our selected plants, very less genetic linkage drag was observed for transfer of *Sub1*, *Xa21*, *xa13*, *xa5 and Xa4* genes (Fig. 9) as the donor parent was an improved genotype already. These improved donor lines IRBB60 and Swarna-Sub1 are likely to contribute less undesirable effects than using a wild or landrace type of donor source for BB durable resistance breeding program. Earlier publications suggest that improved variety as a source of donor was expected to give less undesirable linkage drag compared to the wild and landraces as donors^6,39,40,42^.

A complete recovery of the important phenotypic, grain quality and yield traits of the popular variety Swarna to the pyramided lines along with accumulation of target genes from donor parent was achieved through marker-assisted approach without altering the important traits of the mega variety. Exact Swarna phenotype with in-built durable resistance to BB disease was important for quicker adoption of the converted lines like the recipient variety. The evaluation results indicated that pyramided lines SSB-121-28-13-1, SSB-121-28-13-2, SSB-121-28-13-3, SSB-121-28-13-4, SSB-121-28-13-5, SSB-121-28-13-6, SSB-121-28-13-7, SSB-121-28-13-8 and SSB-121-28-13-9 were yielding higher than recipient variety and also highly similar to the recipient parent ‘Swarna’ (Table 5). Similar results were also reported in earlier publications^5–7,34–37,42^.

Genotype-trait-biplot diagram places the donor parent quite away from the origin and in a separate quadrant from the derived lines. This shows that much less or no drag from donor parent except target genes recovered in the pyramided lines (Fig. 4). However, the recipient parent was placed close to the pyramided lines and in the same quadrant. Thus, the pyramided lines were expected to be similar in phenotype. Many pyramided lines were located near the origin and hence these lines indicated better stability as compared to the distant ones. Evaluation results of 18 pyramided lines for various morpho-quality traits revealed similarity of the lines with the recipient parent while a few were better than the recipient parent for yield, quality and morphologic traits (Figs 7 and 8; Table 5). The genotyping result and its further analysis indicated that the recovery of recipient genome was higher in few backcross derivatives than the theoretical expected value of 75% in backcross generations. In BC_3_F_2_, the recovery of Swarna genome in all homozygous target genes containing derived lines was more than 95 percent (Fig. 6B). The background selection with more number of molecular markers helped in recovery of the recipient genome content in the marker-assisted backcross breeding program. Besides, it revealed that presence of *Sub1* gene with other four BB resistance genes (*Xa21*, *xa13*, *xa5* and *Xa4*) in the same background may not show antagonistic effects for yield and other traits^5,6,36,42,43^.

Evaluation of pyramided lines revealed that lines SSB-121-28-13-1, SSB-121-28-13-2, SSB-121-28-13-3, SSB-121-28-13-4, SSB-121-28-13-5, SSB-121-28-13-6, SSB-121-28-13-7, SSB-121-28-13-8 and SSB-121-28-13-9 exhibited higher yield than the recipient parent Swarna and both the donor parents IRBB60 and Swarna-Sub1. The higher yield obtained might be due to higher level of resistance to BB disease, more tolerance to submergence than the sensitive parent and absence of any yield penalty due to pyramiding of BB resistance genes with *Sub1* QTL in the derived lines. Similar results were also observed in earlier publications^5,6,36,42^. Thus, it created confidence in integrating molecular markers for selection of the desired trait(s) and recovery of the recipient parent’s genome to the derived line in conventional breeding program. Deployment of five genes in a popular variety like Swarna could achieve higher level of tolerance in many BB endemic in lowland rice growing areas of the country. The study clearly establishes the use of marker-assisted selection for conferring resistance/tolerance to biotic and abiotic stresses which is very much important under climatic change situations.

## Materials and Methods

### Plant materials

The donor parent IRBB60, containing BB resistance genes *Xa4*, *xa5*, *xa13* and *Xa21* was used as male parent in the hybridization program with recipient variety ‘Swarna’. The recurrent parent, Swarna is a popular variety of eastern India but highly susceptible to bacterial blight disease and flash flood. Swarna was hybridized with IRBB60 during wet season, 2013 and the true F_1_ plants were backcrossed with third parent Swarna-Sub1 during dry season, 2014 (Fig. 1). During wet season 2014, the true F_1_ plant was again hybridized with recurrent parent ‘Swarna’. Variety Swarna was used as recipient parent than Swarna-Sub1 for better preference by farmers due to its grain coloration, grain quality and ideal maturity duration for shallow lowlands. All 525 BC_1_F_1_ seeds were grown and foreground positive plants for submergence tolerance and four bacterial blight resistance genes were selected by using molecular markers (Table 1). All the foreground positive plants in BC_1_F_1_ generation were subjected to background selection. BC_1_F_1_ line with maximum recurrent genome content was hybridized with Swarna to generate BC_2_F_1_. A Total of 487 BC_2_F_1_ plants were generated during dry season, 2015. The foreground positive plants in BC_2_F_1_ containing maximum recurrent genome content was again hybridized to produce BC_3_F_1_ seeds during wet season, 2015. Background selection was continued in BC_3_F_1_ generation during dry season, 2016. Genotyping was performed to confirm homozygous lines for target gene combinations in BC_3_F_2_ generation. For bioassay and evaluation trial, seeds of the plant carrying homozygous target genes were increased during dry season, 2017 as per the breeding scheme (Fig. 1). Evaluation and bioassay trials were conducted during wet season, 2017 and 2018.

### DNA isolation and PCR amplification

Isolation of mini scale DNA preparation was performed using standard protocol^44^. The PCR reaction mixture contained 30 ng templates DNA, 5 pico mole of each of the primers, 200 μM dNTPs, 1 X PCR buffer (10 mM Tris–HCl, pH 8.3, 50 mM KCl, 1.5 mM MgCl_2_, and 0.01 mg/ml gelatin) and 1 unit of Taq DNA polymerase in a volume of 20 μl and amplification of target sequences were done as per earlier reports (Table 1). The PCR products were separated by gel electrophoresis and imaged on gel documentation system (SynGene, Germany).

### Marker analysis

The publicly available nine tightly linked and gene based markers of the five target genes were used for foreground selection (Table 1). Amongst the 1058 SSRs markers used for parental polymorphism survey, 100 were found to be polymorphic between the parents and used for background selection. Data were analyzed and similarity matrix was constructed from binary data with Jaccard’s coefficients and dendrogram was generated by unweighted pair group method arithmatic average (UPGMA) algorithm, using software^45–47^. Graphical Geno Types (GGT) Version 2.0 software program was used for the assessment of the genomic contribution of the parent in the selected recombinants based on SSR marker data^48^.

### Screening for submergence tolerance

Three weeks’ old seedlings of the BC_3_F_4_ pyramided lines carrying *Sub1* and BB resistance genes along with the three parents were transplanted in the screening tank of ICAR-National Rice Research Institute (NRRI), Cuttack, during wet season, 2017. A population of eighteen plants per row with three rows per entry at 15 × 20 cm spacing was planted in a randomized complete block design with two replications. Complete submergence stress was given for 14 days and water depth was maintained upto 1.5 m each day. After 14 days, the tank was de-submerged and regeneration was noted after a week. Recording of observations and scoring of genotypes were done as described in earlier publication^18^.

### Bioassay against BB resistance

Forty five-day-old pyramided lines carrying BB resistance and *Sub1* genes, along with the control, were inoculated with eight isolates of *Xoo*. Eight highly virulent isolates of BB pathogen identified on their reaction against near isogenic line differentials carrying resistant genes *Xa3*, *Xa4*, *xa5*, *Xa7*, *Xa10*, *xa13* and *Xa21* maintained at ICAR-National Rice Research Institute, Cuttack, Odisha, India were used for inoculation. The reaction of pyramided and parental lines against different *Xoo* inoculated strains were screened during wet season, 2017 and 2018. The *Xoo* isolates were prepared by suspending the bacterial mass in sterile water at a concentration of approximately 10^9^ cells/ml^49^. Five leaves from five different plants of each entry and replication were clip inoculated at the maximum tillering stage and lesion lengths (LL) were recorded after 15 days. The disease symptoms were scored as resistant (R, LL ≤ 3.0 cm), moderately resistant (MR, 3.0 cm < LL ≤ 6.0 cm), moderately susceptible (MS, 6.0 cm < LL ≤ 9.0 cm) or susceptible (S, LL > 9.0 cm) as in the previous publications^6,42,50^.

### Characterization of pyramided lines for morphologic, quality and yield traits

Thirty days old pyramided lines carrying *Sub1* and BB resistance genes and the parents were transplanted in the lowland field. Plot size of 9.6 m^2^ was provided for each entry with forty plants per row and eight rows per entry at 15 × 20 cm spacing in a randomized complete block design with three replications transplanted at NRRI, Cuttack, during wet season, 2017. Data were recorded from ten plants of each entry and replication for agronomic traits *viz*., plant height, panicles/plant, number of filled grains/panicle, 1000-grain weight, grain length, grain breadth, milling (%), head rice recovery (%), kernel length after cooking (mm), alkali spreading value, gel consistency, amylose content (%) while, days to 50% flowering and plot yield were recorded on whole plot basis. Head rice recovery was calculated as per the earlier described method^51^. Estimation of gel consistency (GC) was done as per the standard procedure^52^. Alkali spreading value was computed according to the procedure of^53^. For analysis of cooking qualities, 25 grains were taken in a test tube. Grains were soaked in 20 ml distilled water for 20 minutes after which test tubes were placed in boiling water for 10 min and then cooled. The average length and breadth of 10 cooked kernels were measured. The amylose content of the pyramided lines was estimated as per the standard protocol^54^. Analysis of variance for various agro-morphologic and quality traits and principal component analysis were performed using SAS statistical software^55^.

## Conclusion

Deployment of single resistance gene is risky as there are chances of resistance knock-down by the pathogens. The development of pyramided lines showing higher level of resistance to BB containing four resistance genes namely *Xa21*, *xa13*, *xa5* and *Xa4* along with *Sub1* QTL in the mega variety background is an important achievement for the lowland ecology. The products from the gene stacking workin the ‘Swarna’ background through molecular breeding will provide a solution in the flash-flood and bacterial blight endemic areas of eastern Indian lowland rice ecosystems. The grain and its cooking quality characters *viz*., milling %, head rice recovery %, kernel length (mm), kernel breadth, kernel length after cooking, alkali spreading value, gel consistency and amylose content (%) remained almost same in the pyramided lines as in the recipient parent. The quality features of the mega variety Swarna remained same along with high grain yield and durable bacterial blight resistance in the selected pyramided lines. The diverse agro-climatic zones of our country may offer scope for creation of many virulent *Xoo* strains for which these BB pyramided lines with *Xa21*, *xa13*, *xa5* and *Xa4* along with *Sub1* QTL are expected to provide good substitute to the existing susceptible array of varieties used by the farmers in the targeted region of the country where majority of the areas are rainfed lowlands.

## Supplementary information

Supplementary table S2

Supplementary table S3

## References

[1] Food and Agriculture Organization of the United Nations. *Rice market monitor* 20, 1–38 (2017).

[2] Khush, G. S., Mackill, D. J. & Sidhu, G. S. *Breeding rice for resistance to bacterial leaf blight*. (ed. IRRI) 207–217 (IRRI, Manila, Philippines, 1989).

[3] Sonti RV (1998). Bacterial leaf blight of rice: new insights from molecular genetics. Curr Sci..

[4] Singh S (2001). Pyramiding three bacterial blight resistance genes (xa-5, xa-13 and Xa-21) using marker-assisted selection into indica rice cultivar PR-106. Theor Appl Genet..

[5] Suh JP (2013). Development of breeding lines with three pyramided resistance genes that confer broad-spectrum bacterial blight resistance and their molecular analysis in rice. Rice.

[6] Pradhan SK (2015). Pyramiding of three bacterial blight resistance genes for broad-spectrum resistance in deepwater rice variety, Jalmagna. Rice..

[7] Pradhan SK (2015). Characterization of morpho-quality traits and validation of bacterial blight resistance in pyramided rice genotypes under various hotspots of India. Australian Journal of Crop Science.

[8] Pradhan, S. K. *et al*. Rice research for productivity, profitability and climate resilience (eds Pathak *et al*.) 107–121(NRRI, Cuttack, 2018).

[9] Wu KS, Tanksley SD (1993). Abundance, polymorphism and genetic mapping of micro satellites in rice. Mol Gen Genet.

[10] Yoshimura S (1995). Tagging and combining bacterial blight resistance genes in rice using RAPD and RFLP markers. Mol Breed.

[11] Chen X, Temnykh S, Xu Y, Cho YG, McCouch SR (1997). Development of a microsatellite framework map providing genome-wide coverage in rice (Oryza sativa L.). Theoretical and Applied Genetics.

[12] Rao KK, Lakshminarasu M, Jena KK (2002). DNA markers and marker-assisted breeding for durable resistance to bacterial blight of rice. Biotechnol Adv..

[13] Gu K, Sangha JS, Li Y, Yin Z (2008). High resolution genetic mapping of bacterial blight resistance gene. Xa10. Theor Appl Genet..

[14] Bhasin H (2012). New PCR-based sequence-tagged site marker for bacterial blight resistance gene *Xa38* of rice. Mol Breeding.

[15] Khush GS (2005). What it will take to feed 5.0 billion rice consumers in 2030. Plant Mol Biol..

[16] Ismail AM, Singh US, Singh S, Dar MH, Mackill DJ (2013). The contribution of submergence-tolerant (Sub1) rice varieties to food security in flood prone rainfed lowland areas in. Asia. Field Crops Res..

[17] Iftekharuddaula KM (2012). Rapid and high-precision marker assisted backcrossing to introgress the *SUB1* QTL into BR11, the rainfed lowland rice mega variety of Bangladesh. Euphytica.

[18] Pradhan SK (2015). Comparison of Sub1 markers and their combinations for submergence tolerance and analysis of adaptation strategies of rice in rainfed lowland ecology. Comptes Rendus Biologies.

[19] Bailey-Serres J, Colmer TD (2014). Plant tolerance of flooding stress-recent advances. Plant Cell Environ..

[20] Tamang BG, Fukao T (2015). Plant adaptation to multiple stresses during submergence and following de-submergence. Int. J. Mol. Sci..

[21] Xu K, Mackill DJ (1996). A major locus for submergence tolerance mapped on rice chromosome 9. Mol. Breed..

[22] Xu K, Xu X, Ronald PC, Mackill DJ (2000). A high-resolution linkage map in the vicinity of the rice submergence tolerance locus. Sub1. Mol. Gen. Genet..

[23] Chen M (2002). An integrated physical and genetic map of the rice genome. Plant Cell..

[24] Neeraja CN (2007). A marker-assisted backcross approach for developing submergence-tolerant rice cultivars. Theor Appl Genet..

[25] Manivong P (2014). Marker-assisted selection to improve submergence tolerance, blast resistance and strong fragrance in glutinous rice. Thai J. Genet..

[26] Sridhar R, Reddy JN, Singh UD, Agrawal PK (1999). Usefulness of combinations of bacterial blight resistance genes at Cuttack, Orissa, India. IRRN.

[27] Huang N (1997). Pyramiding of bacterial blight resistance genes in rice: marker assisted selection using RFLP and PCR. Theor Appl Genet..

[28] Sanchez AC, Brar DS, Huang N, Khush GS (2000). Sequence tagged site markers-assisted selection for three bacterial blight resistance genes in rice. Crop Sci..

[29] Shanti ML (2001). Identification of resistance genes effective against bacterial leaf blight pathogen in eastern India. Plant Disease.

[30] Joseph M, Gopalakrishnan S, Sharma RK (2004). Combining bacterial blight resistance and basmati quality characteristics by phenotypic and molecular marker assisted selection in rice. Mol Breed.

[31] Pha PN, Lang NT (2004). Marker assisted selection in rice breeding for bacterial leaf blight. Omon rice.

[32] Bharatkumar S, Paulraj RSD, Brindha PV, Kavitha S, Gnanamanickam SS (2008). Improvent of bacterial blight resistance in rice cultivars ajayothi and IR50 via marker-assisted backcross breeding. J Crop Improve..

[33] Hu KM, Qiu DY, Shen XL, Li XH, Wang SP (2008). Isolation and Manipulation of Quantitative trait Loci for Disease Resistance in Rice Using a Candidate Gene Approach. Mol Plant.

[34] Perez LM, Redona ED, Mendioro MS, Vera Cruz CM, Leung H (2008). Introgression of Xa4, Xa7 and Xa21 for resistance to bacterial blight in thermo-sensitive genetic male sterile rice (Oryza sativa L.) for the development of two-line hybrids. Euphytica.

[35] Sundaram RM (2008). Marker assisted introgression of bacterial blight resistance in Samba Mahsuri, an elite indica rice variety. Euphytica.

[36] Dokku P, Das KM, Rao GJN (2013). Pyramiding of four resistance genes of bacterial blight in Tapaswini, an elite rice cultivar, through marker-assisted selection. Euphytica.

[37] Rajpurohit D (2011). Pyramiding of two bacterial blight resistance and a semi dwarfing gene in Type 3 basmati using marker-assisted selection. Euphytica.

[38] Narayanan NN (2004). Molecular breeding: Marker-assisted selection combined with biolistic transformation for blast and bacterial blight resistance in *indica* rice (cv. CO39). Mol Breeding.

[39] Das G, Rao GJN (2015). Molecular marker assisted gene stacking for biotic and abiotic stress resistance genes in an elite rice cultivar. Front. Plant Sci..

[40] Das (2018). Improved Tapaswini having four BB resistance genes pyramided with six genes/QTLs, resistance/tolerance to biotic and abiotic stresses in rice. Scientific report..

[41] Han X (2014). Quantitative Trait Loci Mapping for Bacterial Blight Resistance in Rice Using Bulked Segregant Analysis. Int. J. Mol. Sci..

[42] Pradhan (2016). Incorporation of bacterial blight resistance genes into lowland rice cultivar through marker assisted backcross breeding. Phytopathology.

[43] Sundaram, R. M. *et al*. Genomics and Crop Improvement: Relevance and Reservations (eds Muralidharan, K. & Siddiq, E.A.) 154–182 (Acharya NG Ranga Agricultural University, Hyderabad, India, 2011).

[44] Dellaporta SL, Wood J, Hicks JB (1983). A plant DNA mini preparation: version II. Plant Mol Biol Rep..

[45] Pavalíce A, Hrda S, Flegr J (1999). Free Tree—freeware program for construction of phylogenetic trees on the basis of distance data and bootstrap/jackknife analysis of the tree robustness, Application in the RAPD analysis of genus Frenkelia. Folia Biol. (Praha).

[46] Hampl V, Pavlicek A, Flegr J (2001). Construction and bootstrap analysis of DNA fingerprinting based phylogenetic trees with the freeware program FreeTree: application to trichomonad parasites. Intl J Syst Evol Microbiol..

[47] Page RD (1996). TreeView: an application to display phylogenetic trees on personal computers. Comput Appl Biosci.

[48] Van Berloo R (1999). GGT: software for display of graphical genotypes. J Hered.

[49] Kauffman HE, Reddy APK, Hsien SPY, Merca SD (1973). An improved technique for evaluating resistance of rice varieties to *Xanthomonas oryzae*. Plant Dis Report.

[50] Amante-Bordeos A (1992). Transfer of bacterial blight and blast resistance from the tetraploid wild rice Oryza minuta to cultivated rice, Oryza sativa. Theor Appl Genet..

[51] Tan YF (1999). The three important traits for cooking and eating quality of rice grains are controlled by a single locus in an elite rice hybrid, Shanyou 63. Theor Appl Genet..

[52] Cagampang GB, Perez CM, Juliano BO (1973). A gel consistency test for eating quality in rice. J Sci Food Agr..

[53] Little RR, Hilder GB, Dawson EH (1958). Differential effect of dilute alkali on 25 varieties of milled white rice. Cereal Chem..

[54] Juliano, B.O. Rice quality screening with the Rapid ViscoAnalyser. (eds Walker, C. E. & Hazelton, J. L.) 19–24 (Newport Scientific, Sydney, 1996).

[55] SAS Institute Inc. SAS ® 9.2 Language Reference: Concepts, (ed. Second) 626–978 (SAS Institute Inc. USA, Cary, NC, 2010).

[56] Chu Z (2006). Targeting *xa13*, a recessive gene for bacterial blight resistance in rice. Theor Appl Genet..

[57] Hajira S (2014). Development durable bacterial blight resistant lines of Samba Mahsuri possessing Xa33, Xa21, xa13 & xa5. Progressive Research.

[58] Ma BJ (1999). Studies of PCR marker for the rice bacterial blight resistance gene Xa-4. Hereditas.

[59] Pandit E (2017). Genome-wide association mapping reveals multiple QTLs governing tolerance response for seedling stage chilling stress in *indica* rice. Frontiers in plant science.

