# Supplementary material for: Development of flash-flood tolerant and durable bacterial blight resistant versions of mega rice variety ‘Swarna’ through marker-assisted backcross breeding
Source: Sci Rep. 2019 Sep 5;9:12810. doi: 10.1038/s41598-019-49176-z (PMC6728354; doi:10.1038/s41598-019-49176-z)
Supplement: Supplementary file 1 — Supplementary table S2 [file 41598_2019_49176_MOESM1_ESM.doc]

**Development of flash-flood tolerant and durable bacterial blight resistant versions of mega rice variety ‘Swarna’ through marker-assisted backcross breeding**

**Sharat Kumar Pradhan^1†^, Elssa Pandit^1†^, Swapnil Pawar^1^, Shaikh Yasin Baksh^1^, Arup Kumar Mukherjee^2^ & Shakti Prakash Mohanty^1^**

^†^ Authors contributed equally to this experiment

**Supplementary Table S2**. Bacterial blight disease score and reaction of pyramided and parental lines against different *Xoo* inoculated strains during wet season, 2017

| Sl.  No. | Pyramided lines | Gene combination |  | Mean lesion length (MLL) in cm (Mean±standard error) | | | | | | | | | |
| --- | --- | --- | --- | --- | --- | --- | --- | --- | --- | --- | --- | --- | --- |
|  |  |  | *Xoo* strains inoculated | | | | | | | | | | Disease reaction |
|  |  |  | Xa-17 | | Xa-7 | xa-2 | xb-7 | xc-4 | xd-1 | xa-1 | xa-5 | MLL |  |
| 1 | SSB-121-28-13-1 | Xa21+xa13+xa5+Xa4 | 2.2±0.25 | | 2.4±0.32 | 2.5±0.52 | 2.4±0.75 | 2.6±0.68 | 2.9±0.76 | 2.3±0.82 | 2.3±0.85 | 2.45 | R |
| 2 | SSB-121-28-13-2 | Xa21+xa13+xa5+Xa4 | 2.8±0.73 | | 2.9±0.86 | 2.6±1.04 | 2.7±0.84 | 2.7±0.72 | 2.8±0.67 | 2.5±0.83 | 2.7±0.95 | 2.71 | R |
| 3 | SSB-121-28-13-3 | Xa21+xa13+xa5+Xa4 | 2.6±0.92 | | 2.3±0.54 | 2.8±0.91 | 2.9±0.48 | 2.8±0.76 | 2.6±0.82 | 1.8±1.12 | 1.9±1.05 | 2.46 | R |
| 4 | SSB-121-28-13-4 | Xa21+xa13+xa5+Xa4 | 2.2±0.72 | | 2.6±0.94 | 2.7±0.69 | 3.1±0.43 | 3.2±0.42 | 2.4±0.72 | 2.8±0.66 | 2.3±0.74 | 2.66 | R |
| 5 | SSB-121-28-13-5 | Xa21+xa13+xa5+Xa4 | 2.5±0.64 | | 3.1±0.53 | 2.6±0.72 | 2.1±0.54 | 3.1±0.64 | 2.3±0.82 | 1.9±1.24 | 2.7±0.76 | 2.54 | R |
| 6 | SSB-121-28-13-6 | Xa21+xa13+xa5+Xa4 | 1.7±0.72 | | 2.3±0.58 | 2.7±0.85 | 2.3±0.59 | 2.3±0.92 | 2.7±0.85 | 3.1±0.42 | 2.3±0.91 | 2.43 | R |
| 7 | SSB-121-28-13-7 | Xa21+xa13+xa5+Xa4 | 3.2±0.58 | | 2.7±0.68 | 2.7±0.63 | 2.5±0.88 | 2.7±0.84 | 1.9±0.78 | 4.1±1.25 | 2.3±0.46 | 2.76 | R |
| 8 | SSB-121-28-13-8 | Xa21+xa13+xa5+Xa4 | 3.3±0.82 | | 3.1±0.96 | 2.1±0.95 | 2.3±0.77 | 2.6±0.74 | 2.5±0.82 | 2.7±0.76 | 2.4±0.71 | 2.63 | R |
| 9 | SSB-121-28-13-9 | Xa21+xa13+xa5+Xa4 | 2.9±0.84 | | 2.3±0.74 | 2.5±1.35 | 2.5±0.82 | 3.1±1.32 | 2.0±0.88 | 2.7±0.86 | 2.9±0.83 | 2.61 | R |
| 10 | SSB-121-28-13-10 | Xa21+xa13+xa5+Xa4 | 3.1±0.86 | | 2.4±0.78 | 3.2±0.80 | 3.3±0.62 | 2.6±0.42 | 2.8±0.76 | 2.7±0.58 | 3.3±0.66 | 2.95 | R |
| 11 | SSB-121-28-13-11 | Xa21+xa13+xa5+Xa4 | 3.3±0.96 | | 2.3±0.64 | 3.2±0.96 | 2.4±0.52 | 2.3±0.52 | 2.5±0.69 | 2.9±0.36 | 3.4±0.59 | 2.79 | R |
| 12 | SSB-121-28-13-12 | Xa21+xa13+xa5+Xa4 | 2.8±0.64 | | 2.3±0.36 | 3.5±0.86 | 2.9±0.62 | 2.7±0.44 | 2.7±0.72 | 2.4±0.81 | 2.8±0.89 | 2.76 | R |
| 13 | SSB-121-28-13-13 | Xa21+xa13+xa5+Xa4 | 3.2±0.83 | | 3.1±1.14 | 1.6±0.28 | 1.6±0.42 | 2.5±0.71 | 2.7±0.68 | 2.4±0.47 | 1.9±0.25 | 2.38 | R |
| 14 | SSB-121-28-13-14 | Xa21+xa13+xa5+Xa4 | 1.8±0.58 | | 2.7±0.54 | 1.7±0.52 | 2.9±0.78 | 2.2±0.46 | 1.6±0.22 | 3.6±1.42 | 1.6±0.39 | 2.26 | R |
| 15 | SSB-121-28-13-15 | Xa21+xa13+xa5+Xa4 | 2.6±0.76 | | 2.9±1.04 | 1.8±0.92 | 2.8±0.78 | 2.7±0.72 | 3.3±1.16 | 2.2±0.56 | 3.9±1.24 | 2.78 | R |
| 16 | SSB-121-28-13-16 | Xa21+xa13+xa5+Xa4 | 2.9±0.89 | | 2.6±0.78 | 2.4±0.68 | 2.9±0.88 | 3.1±1.23 | 3.3±0.84 | 2.2±0.92 | 2.7±0.83 | 2.76 | R |
| 17 | SSB-121-28-13-17 | Xa21+xa13+xa5+Xa4 | 4.1±0.62 | | 4.3±0.84 | 3.1±0.52 | 3.4±0.78 | 5.2±0.98 | 4.7±0.68 | 5.2±0.82 | 4.3±0.96 | 4.33 | MR |
| 18 | SSB-121-28-13-18 | Xa21+xa13+xa5+Xa4 | 5.2±0.78 | | 3.4±0.78 | 4.6±0.92 | 3.8±1.18 | 3.9±0.38 | 4.3±0.58 | 4.5±0.72 | 4.6±0.74 | 4.29 | MR |
| 19 | IRBB60 (donor) | Xa21+xa13+xa5+Xa4 | 1.8±0.53 | | 2.6±0.46 | 2.7±0.58 | 2.3±0.39 | 2.9±0.46 | 2.7±0.48 | 2.8±0.92 | 2.4±0.41 | 2.53 | R |
| 20 | Swarna-Sub1 | - | 12.8±1.24 | | 13.1±1.35 | 13.4±1.45 | 11.6±0.96 | 10.3±1.22 | 9.6±0.42 | 10.3±0.52 | 12.6±0.76 | 11.71 | S |
| 21 | Swarna (recipient) | - | 13.4±1.65 | | 12.6±1.76 | 12.2±1.48 | 10.4±1.34 | 9.8±1.38 | 10.6±1.74 | 14.0±1.78 | 13.7±1.82 | 12.19 | S |

R: Resistant; MR: Moderately resistant; S: Susceptible; MLL: Mean lesion length in cm
