# Supplementary material for: Development of flash-flood tolerant and durable bacterial blight resistant versions of mega rice variety ‘Swarna’ through marker-assisted backcross breeding
Source: Sci Rep. 2019 Sep 5;9:12810. doi: 10.1038/s41598-019-49176-z (PMC6728354; doi:10.1038/s41598-019-49176-z)
Supplement: Supplementary file 2 — Supplementary table S3 [file 41598_2019_49176_MOESM2_ESM.doc]

**Development of flash-flood tolerant and durable bacterial blight resistant versions of mega rice variety ‘Swarna’ through marker-assisted backcross breeding**

**Sharat Kumar Pradhan^1†^, Elssa Pandit^1†^, Swapnil Pawar^1^, Shaikh Yasin Baksh^1^, Arup Kumar Mukherjee^2^ & Shakti Prakash Mohanty^1^**

^†^ Authors contributed equally to this experiment

**Supplementary Table S3**. Bacterial blight Bacterial blight disease score and reaction of pyramided and parental lines against different *Xoo* inoculated strains during wet season, 2018

| Sl.  No. | Pyramided lines | Gene combination |  | | Mean lesion length (MLL) in cm (Mean±standard error) | | | | | | | | |
| --- | --- | --- | --- | --- | --- | --- | --- | --- | --- | --- | --- | --- | --- |
|  |  |  | *Xoo* strains inoculated | | | | | | | | | | Disease reaction |
|  |  |  | Xa-17 | Xa-7 | | xa-2 | xb-7 | xc-4 | xd-1 | xa-1 | xa-5 | MLL |  |
| 1 | SSB-121-28-13-1 | Xa21+xa13+Xa4 | 2.8±0.41 | 2.6±0.39 | | 3.5±0.61 | 2.9±0.65 | 3.2±0.73 | 3.2±0.68 | 2.5±0.72 | 2.6±0.76 | 2.91±0.68 | R |
| 2 | SSB-121-28-13-2 | Xa21+xa13+xa5+Xa4 | 3.1±0.76 | 2.7±0.76 | | 2.4±0.94 | 2.8±0.87 | 2.8±0.75 | 2.9±0.72 | 2.6±0.71 | 2.5±0.84 | 2.72±0.80 | R |
| 3 | SSB-121-28-13-3 | Xa21+xa13+Xa4 | 3.2±0.96 | 2.8±0.57 | | 2.9±0.96 | 3.2±0.75 | 2.9±0.71 | 2.8±0.85 | 2.7±1.03 | 2.6±0.76 | 2.88±0.84 | R |
| 4 | SSB-121-28-13-4 | Xa21+xa13+Xa4 | 3.3±0.81 | 2.9±0.83 | | 3.1±0.78 | 3.2±0.84 | 3.3±0.52 | 3.4±0.85 | 2.9±0.81 | 2.7±0.63 | 3.1±0.74 | MR |
| 5 | SSB-121-28-13-5 | Xa21+xa13+Xa4 | 2.8±0.73 | 3.2±0.68 | | 2.9±0.78 | 2.6±0.65 | 3.0±0.53 | 2.7±0.86 | 2.6±0.92 | 2.9±0.79 | 2.8±0.75 | R |
| 6 | SSB-121-28-13-6 | Xa21+xa13+Xa4 | 2.6±0.61 | 2.5±0.47 | | 2.8±0.81 | 2.7±0.67 | 2.9±0.83 | 3.2±0.74 | 2.9±0.57 | 2.8±0.74 | 2.8±0.72 | R |
| 7 | SSB-121-28-13-7 | Xa21+xa13+xa5+Xa4 | 2.9±0.65 | 2.8±0.62 | | 3.1±0.69 | 2.7±0.75 | 2.6±0.73 | 2.5±0.69 | 3.2±0.93 | 2.6±0.54 | 2.8±0.72 | R |
| 8 | SSB-121-28-13-8 | Xa21+xa13+Xa4 | 3.5±1.03 | 3.4±0.92 | | 2.5±0.83 | 2.7±0.81 | 2.9±0.62 | 3.1±0.76 | 3.10±0.76 | 3.2±0.85 | 3.05±0.78 | MR |
| 9 | SSB-121-28-13-9 | Xa21+xa13+Xa4 | 3.2±0.73 | 2.8±0.54 | | 2.9±0.72 | 2.8±0.64 | 3.2±1.08 | 2.7±0.81 | 2.8±0.79 | 3.0±0.74 | 2.92±0.79 | R |
| 10 | SSB-121-28-13-10 | Xa21+xa13+xa5+Xa4 | 2.9±0.63 | 2.7±0.55 | | 3.1±0.75 | 3.2±0.68 | 2.8±0.56 | 3.1±0.82 | 2.9±0.62 | 3.2±0.84 | 2.98±0.71 | R |
| 11 | SSB-121-28-13-11 | Xa21+xa13+Xa4 | 3.5±1.02 | 2.8±0.78 | | 3.3±0.85 | 2.8±0.56 | 2.9±0.64 | 3.2±0.81 | 3.1±0.55 | 2.8±0.71 | 3.35±0.86 | MR |
| 12 | SSB-121-28-13-12 | Xa21+xa13+Xa4 | 3.4±0.69 | 3.5±0.75 | | 3.6±0.92 | 3.7±0.74 | 3.4±0.75 | 3.5±0.93 | 3.2±0.96 | 3.1±0.84 | 3.35±0.85 | MR |
| 13 | SSB-121-28-13-13 | Xa21+xa13+Xa4 | 3.4±0.91 | 3.5±1.05 | | 3.7±0.82 | 3.5±0.96 | 2.9±0.65 | 3.2±0.76 | 3.6±0.94 | 3.4±1.12 | 3.4±0.88 | MR |
| 14 | SSB-121-28-13-14 | Xa21+xa13+Xa4 | 3.2±0.75 | 2.6±0.48 | | 3.2±0.55 | 3.7±0.66 | 2.5±0.63 | 2.7±0.51 | 3.2±0.83 | 2.9±0.68 | 3.0±0.63 | R |
| 15 | SSB-121-28-13-15 | Xa21+xa13+Xa4 | 2.8±0.52 | 2.6±0.73 | | 2.8±0.54 | 2.5±0.55 | 2.9±0.62 | 3.1±0.91 | 2.6±0.49 | 3.8±1.13 | 2.88±0.70 | R |
| 16 | SSB-121-28-13-16 | Xa21+xa13+Xa4 | 3.2±0.82 | 2.9±0.66 | | 3.1±0.55 | 3.2±0.77 | 3.4±0.86 | 3.5±0.77 | 2.8±0.85 | 2.9±0.76 | 3.12±0.76 | MR |
| 17 | SSB-121-28-13-17 | Xa21+xa13+Xa4 | 4.3±0.75 | 4.2±0.55 | | 3.7±0.71 | 3.8±0.69 | 4.7±0.72 | 4.5±0.83 | 5.1±0.68 | 4.7±0.74 | 4.37±0.73 | MR |
| 18 | SSB-121-28-13-18 | Xa21+xa13+Xa4 | 5.4±0.83 | 3.8±0.81 | | 4.3±0.85 | 4.2±0.69 | 4.1±1.12 | 4.6±0.86 | 4.7±0.49 | 5.3±0.88 | 4.55±0.82 | MR |
| 19 | IRBB60 (donor) | Xa21+xa13+Xa4 | 2.3±0.44 | 2.5±0.52 | | 2.1±0.61 | 2.8±0.54 | 3.1±1.13 | 2.6±0.56 | 2.9±0.72 | 2.5±0.68 | 2.60±0.70 | R |
| 20 | Swarna-Sub1 | - | 11.3±1.35 | 12.4±1.14 | | 11.3±1.04 | 10.2±1.08 | 10.7±1.17 | 10.5±0.89 | 10.7±1.23 | 10.4±0.98 | 10.93±1.07 | S |
| 21 | Swarna(recipient) | - | 12.6±1.16 | 12.9±1.12 | | 12.7±1.31 | 11.5±1.13 | 10.3.±1.15 | 11.8±1.44 | 13.4±1.62 | 13.4±1.45 | 12.37±1±.34 | S |
